# Survival on Home Dialysis in New Zealand

**DOI:** 10.1371/journal.pone.0096847

**Published:** 2014-05-07

**Authors:** Mark R. Marshall, Rachael C. Walker, Kevan R. Polkinghorne, Kelvin L. Lynn

**Affiliations:** 1 Faculty of Medical and Health Sciences, University of Auckland, Auckland, New Zealand; 2 Department of Renal Medicine, Counties Manukau District Health Board, Auckland, New Zealand; 3 Australia and New Zealand Dialysis and Transplant Registry (ANZDATA), The Royal Adelaide Hospital, Adelaide, South Australia, Australia; 4 Renal Department, Hawke’s Bay District Health Board, Hastings, New Zealand; 5 Sydney School of Public Health, University of Sydney, Sydney, New South Wales, Australia; 6 Department of Nephrology, Monash Medical Centre, Clayton, Victoria, Australia; 7 Departments of Medicine and Epidemiology and Preventive Medicine, Monash University, Clayton, Victoria, Australia; 8 Kidney Health New Zealand, Christchurch, New Zealand; University of Sao Paulo Medical School, Brazil

## Abstract

**Background:**

New Zealand (NZ) has a high prevalence of both peritoneal dialysis (PD) and home haemodialysis (HD) relative to other countries, and probably less selection bias. We aimed to determine if home dialysis associates with better survival than facility HD by simultaneous comparisons of the three modalities.

**Methods:**

We analysed survival by time-varying dialysis modality in New Zealanders over a 15-year period to 31-Dec-2011, adjusting for patient co-morbidity by Cox proportional hazards multivariate regression.

**Results:**

We modelled 6,419 patients with 3,254 deaths over 20,042 patient-years of follow-up. Patients treated with PD and facility HD are similar; those on home HD are younger and healthier. Compared to facility HD, home dialysis (as a unified category) associates with an overall 13% lower mortality risk. Home HD associates with a 52% lower mortality risk. PD associates with a 20% lower mortality risk in the early period (<3 years) that is offset by a 33% greater mortality risk in the late period (>3 years), with no overall net effect. There was effect modification and less observable benefit associated with PD in those with diabetes mellitus, co-morbidity, and in NZ Maori and Pacific People. There was no effect modification by age or by era.

**Conclusion:**

Our study supports the culture of home dialysis in NZ, and suggests that the extent and duration of survival benefit associated with early PD may be greater than appreciated. We are planning further analyses to exclude residual confounding from unmeasured co-morbidity and other sociodemographic factors using database linkage to NZ government datasets. Finally, our results suggest further research into the practice of PD in NZ Maori and Pacific People, as well as definitive study to determine the best timing for switching from PD in the late phase.

## Introduction

In New Zealand (NZ), there is general consensus that dialysis at home is preferred to dialysis at medical facilities for clinically appropriate people with end-stage kidney disease (ESKD) [1]. This is driven by clinical, patient-centred and health delivery considerations. Research and cumulative clinical experience both point to better survival and patient experience for those dialysing at home compared to those dialysing in facilities [2]–[6]. Economic evaluations consistently demonstrate lower cost for home dialysis compared to facility dialysis [7], [8]. Driven by these factors, there has been a longstanding culture of promoting home dialysis in NZ, resulting in a prevalence that is higher than all but one of the other Organisation for Economic Co-operation and Development (OECD) nations [9].

Globally, there is a resurgence of interest in home dialysis, driven by the identifiable benefits and also the need for cost containment in the context of unstable global economic conditions [10]–[12]. A key issue for those promoting home dialysis is the validity and extent of the assertion of better outcomes with home dialysis. This assertion is founded on a range of observational analyses that are biased by a number of issues, the most significant being selection bias. In this regard, NZ is a valuable setting for repeating these analyses, due to its uniquely high prevalence of both home HD and PD. This situation usually translates to improved internal validity through less selection bias, and provides sufficient statistical power to allow simultaneous comparisons of all modalities including home HD. In addition, NZ is a economically developed nation with results that are reasonably generalizable. In this research, we address the following clinical question: In a country such as NZ, is home dialysis independently associated with better survival than facility haemodialysis (HD), accounting for patient characteristics?

We present analyses in a restricted cohort of New Zealanders from the Australian and New Zealand Dialysis and Transplant Registry (ANZDATA) Registry which has prospectively collected data on ESKD patients in these countries since 1963 (www.anzdata.org.au). We compare survival over a 15-year period between those treated with home dialysis and those treated with facility HD, adjusting for measured patient risk factors such as age and co-morbidity.

## Methods

### Study Design

We performed a cohort study, using an as-treated framework (“did the exposure that the patient actually receive affect mortality?”), as opposed to an intention-to-treat framework (“did exposure that the patient initially receive affect mortality, irrespective of subsequent changes that occurred along the way?”) [13]. The study protocol was reviewed and approved by the National (NZ) Health and Disability Ethics Committee (IORG0000895) and the Counties Manukau Institutional Review Board, and the need for patient consent waived under the provisions for retrospective audit and observational study.

### Participants and Data Source

The ANZDATA Registry collects data on all ESKD patients from every dialysis centre in Australia and NZ. ESKD patients are defined as those with a diagnosis of chronic kidney disease, and for whom renal replacement therapy is intended to be an indefinite treatment. Until 2004, data collection rounds occurred at 6-monthly intervals (31 March and 30 September). Since 2004, rounds have occurred annually (31 December). Details of the structure and methods of the registry have been reported elsewhere [14].

We created an inception cohort of adult ESKD patients (≥18 years at dialysis inception), whose first episode renal replacement therapy occurred after 1-Jan-1997 in NZ. Prevalent patients at the start of the period of observation were not included, a measure which avoids lead time bias, and immortal time bias. Patients were followed through their entire patient journey until death or 31-December-2011, whichever occurred first. Censoring was at the time of kidney transplantation, return of renal function or loss to follow-up.

### Primary Exposure and Outcome Variables

The primary exposure in this study is time-varying dialysis modality. The exposure of ‘facility HD’ was defined as dialysis in a dependent fashion at a staffed hospital or satellite dialysis HD unit, and the exposure of ‘home dialysis’ was defined as dialysis in an independent fashion in an unstaffed setting using either HD or peritoneal dialysis (PD). Home dialysis was further analysed in separate categories of PD or home HD. We did not further subcategorize HD modalities as being frequent or extended hours: a previous study in Australia and NZ has shown mortality benefit with home HD and its related characteristics, but not with frequent/extended operating characteristics per se [4].

The primary outcome was patient mortality, based upon details provided by the treating nephrologist.

### Data Measurement and Quantitative Variables

We adjusted for known patient-related risk factors collected in the ANZDATA Registry: age, gender, ethnicity, primary kidney disease, estimated glomerular filtration rate (eGFR) [15], late referral for nephrology pre-dialysis care (<3 months before dialysis inception), diabetes mellitus (none, type 1, type 2), body mass index (BMI), medical co-morbidity (coronary artery disease, peripheral vascular disease, cerebrovascular disease, chronic lung disease), smoking, plasma hemoglobin, and year of dialysis inception to account for any secular variation. We did not include serum calcium/phosphate, since these data have only been collected since 1 October 2003. Medical co-morbidity was modeled as time-varying, according to presence or absence at the point of each modality change.

### Statistical Methods

We constructed exposure-outcome models for survival using Cox proportional hazards regression. For all comparative analyses of mortality risk by modality, we modeled facility HD as the reference category. In the first set of analyses, we compared facility HD with home dialysis, and in the second set we compared facility HD with PD and home HD separately. Modality was modelled as time varying, and handled by episode-splitting within patients, assuming a constant hazard rate within time periods of modality.

We used two and three way interaction terms in the main-effects models to test effect modification by age, ethnicity, comorbidity, BMI, the presence and type of diabetes mellitus, and year of dialysis inception. We chose these interactions as being clinically plausible, on the basis of both published literature as well as cumulative clinical experience. Interactions were assessed using the two-tailed Wald test P values as a guide to selecting interaction terms for testing, with significance within the model using the likelihood ratio test (P value <0.05). Where effect modification was evinced, separate models were developed in each subgroup.

Continuous co-variates such as age and BMI were modeled as quantiles in order to avoid the assumption of linear relationships. We removed co-variates from the multivariate model in a backward stepwise fashion beginning with the co-variate with the highest P value from two-tailed Wald tests of the individual coefficients, using the partial likelihood ratio test to compare the new reduced model with the older larger. We based final confounder selection upon both biological plausibility and contribution to the comprehensibility of the model, and also the significance of the co-variate within the model as assessed by the two-tailed partial likelihood ratio test P value at a level of <0.2 when jointly adjusted for other covariates.

The findings of this study are largely expected and confirmatory, although there are novel findings. We re-iterate that our facility HD and PD populations were very balanced in terms of demographics and co-comorbidity, and that very few studies in the literature report outcomes that compare all three dialysis modalities (facility HD, PD and home HD). In our study, there are sufficient data-points for us to estimate the effects of facility HD and home HD separately, and treat the exposures separately. As such, our data allow us demonstrate the extended duration of the survival advantage with PD compared to facility HD, with perhaps more confidence than other investigators. In addition, this also allows us to directly compare PD (10880 records, 3738 patients, 1525 failures) with home HD (1942 records, 1942 patients, 194 failures) as sub-modalities of home dialysis. In our study, it is clear that home HD is associated with the lowest mortality risk of the home modalities, even within the first 3 years: the a hazard ratio for death (95% confidence intervals) with home HD is 0.52 (0.41–0.65) compared to PD in the early period.

We included a gamma distributed shared frailty, using the center of initial dialysis treatment as the group. This procedure adds a random effect to model heterogeneity of effect across defined groups, and accounts in this case for the ‘treatment-by-centre’ interaction. We included a shared frailty in the final model based upon significance at a level of <0.05 as assessed by the two-tailed partial likelihood ratio test.

The assumption for proportional hazards for the final models was assessed formally by the use of scaled Schoenfeld residuals, and visually by -ln [-ln(survival)] versus ln(analysis time) plots for modality, adjusted for confounders. We assessed overall goodness of fit visually by comparing plots of Kaplan-Meier observed survival curves to the Cox predicted curves for modality.

Where necessary, comparisons between groups were made using the Student’s t-test, Mann–Whitney U or Kruskal–Wallis tests, or chi-square test as appropriate.

Statistical significance to associations was attributed to findings if the two-tailed P value was <0.05.

We used Stata Intercooled MP/11.2 for analyses (StataCorp, www.stata.com).

## Results

### Participants and Outcome Data

We identified an inception cohort of 6483 adult patients with 3298 deaths over 20,123 patient-years follow-up. There were 6419 with 3254 deaths over 20,042 patient-years of follow-up with sufficient data for modelling: Table 1 summarizes the inception and study cohorts. Of note, those with missing data had a higher probability of being late referrals with a low eGFR at dialysis inception, active smokers, and diabetes mellitus. Of the modelled deaths, 52% were due to cardiovascular causes, 14% due to infectious causes, and the remainder due to treatment withdrawal, cancer and other causes.

**Table 1:** Clinical characteristics of the inception cohort.

Variable		Study Cohort	Excluded Cohort withMissing Data
Number		6,419	64
Age	Years	59.2 (49.3, 68.1)	60.6 (51.5, 66.1)
Gender	Male	3,838 (59.2)	40 (62.5)
	Female	2,581 (40.2)	24 (37.5)
Ethnicity	NZ European-&-Other	2,921 (45.5)	30 (46.9)
	NZ Maori	2,053 (32)	28 (43.8)
	Asian	378 (5.9)	1 (1.6)
	Pacific People	1,067 (16.6)	5 (7.8)
Late referral*		1,486 (23.1)	26 (35.9)
eGFR*	mL/min/1.73 m^2^	6.2 (4.3, 8.8)	4.7 (0,7.5)
Smoking*		1,073 (16.7)	11 (17.2)
Diabetes Mellitus*	Type 1	220 (3.4)	3 (4.7)
	Type 2	2,997 (46.7)	36 (56.3)
Primary renal	Glomerulonephritis/Other	2,797 (43.6)	22 (34.4)
Disease	Hypertension/Ischemic	743 (11.6)	13 (20.3)
	Diabetic nephropathy	2,879 (44.9)	29 (45.3)
Co-morbid disease	Coronary artery	2,422 (37.7)	30 (46.9)
at baseline	Peripheral vascular*	1,528 (23.8)	27 (42.2)
	Cerebrovascular	893 (13.8)	13 (20.3)
	Lung	1,111 (17.3)	17 (26.6)
Co-morbid disease	Coronary artery	2,871 (44.7)	32 (50)
at end of follow-up	Peripheral vascular*	1,850 (28.8)	29 (45.3)
	Cerebrovascular	1,068 (16.6)	13 (20.3)
	Lung	1,279 (19.9)	18 (28.1)
Body mass index*	kg/m^2^	28.1 (24.2, 3.8)	33.3 (25.5, 36.4)

***Note***: Continuous variables are shown as median (25th, 75th percentile); categorical variables are shown are number (percentage). For the excluded cohort with missing data, the sum of patients within each category equals the number of patients of within this cohort who have data pertaining to that category.

*P<0.05 for study cohort versus excluded cohort with missing data.

### Descriptive Data

Table 2 summarizes patient characteristics of the study cohort at modality inception (the point of commencement of dialysis modality). In this table only, some patients are classified in multiple modality categories according to their time-varying exposure over the period of observation, although for Table 1 and all analyses of mortality by modality every patient is “counted” only once. Patients treated with facility HD and PD had comparable demographics and co-morbidity. Compared to those treated with facility HD or PD, however, patients on home HD were younger, more likely to have ESKD secondary to single-organ (e.g. glomerulonephritis) rather than systemic disease, and less likely to have diabetes mellitus or medical co-morbidity.

**Table 2:** Clinical characteristics of the study cohort at modality inception.

Variable		FacilityHemodialysis	PeritonealDialysis	HomeHemodialysis	HomeDialysis
Number		8.713	9,728	1,547	11,275
Age* #	Years	57.3 (47.6, 65.9)	59.8 (50.2, 68.5)	50.7 (41.5, 59.3)	58
Gender* #	Male	5,258 (60.3)	5,412 (55.6)	1,061 (68.6)	6,473 (57.4)
	Female	3,455 (39.7)	4,316 (44.4)	486 (31.4)	4,802 (42.6)
Ethnicity* #	NZ European-&-Other	3, 169 (36.4)	4,590 (47.2)	748 (48.4)	5,338 (47.3)
	NZ Maori	3,068 (35.2)	3,309 (34)	542 (35)	3,3851 (34.2)
	Asian	465 (5.3)	611 (6.3)	57 (3.7)	688 (5.9)
	Pacific People	2,011 (23.08)	1,218 (12.5)	200 (12.9)	1,418 (12.6)
Late referral* #		2,310 (26.5)	2,293 (23.6)	290 (18.8)	2,583 (22.9)
eGFR* #	mL/min/1.73 m^2^	5.8 (4.1, 8.2)	6.5 (4.5, 9.1)	5.7 (3.8, 7.6)	6.4 (4.4, 8.9)
Smoking		1,537 (17.6)	1,672 (17.2)	264 (17.1)	1,936 (17.2)
Diabetes Mellitus* #	Type 1	246 (2.8)	404 (4.3)	46 (2.8)	450 (4.0)
	Type 2	4,378 (50.3)	4,381 (45)	537 (34.7)	4,918 (43.6)
Primary renal* #	Glomerulonephritis/Other	3,694 (42.4)	4,106 (42.2)	918 (59.3)	5,024 (44.6)
Disease	Hypertension/Ischemic	846 (9.71)	1,259 (12.9)	127 (8.2)	1,386 (12.3)
	Diabetic nephropathy	4,173 (47.9)	4.363 (44.9)	502 (32.5)	4,865 (43.1)
Co-morbid disease	Coronary artery* #	3,521 (40.5)	3,950 (40.6)	453 (29.3)	4,403 (39.1)
at baseline	Peripheral vascular*	2,236 (25.7)	2,645 (27.2)	232 (15)	2,877 (25.5)
	Cerebrovascular*	1,251 (14.4)	1,538 (15.8)	108 (7)	1,646 (14.6)
	Lung* #	1,713 (19.7)	1,684 (17.3)	231 (14.9)	1,915 (17.0)
Co-morbid disease	Coronary artery* #	4,118 (47.3)	4,573 (47)	550 (35.6)	5,123 (45.4)
at end of follow-up	Peripheral vascular*	2,633 (30.2)	3,151 (32.4)	298 (19.3)	3,449 (30.6)
	Cerebrovascular*	1,454 (16.7)	1,848 (19)	136 (8.8)	1,984 (17.6)
	Lung* #	1,965 (22.6)	1,894 (19.5)	277 (17.9)	2,171 (19.3)
Body mass index*	kg/m^2^	29.0 (24.8, 34.2)	27.3 (24, 31.1)	29.7 (25.4, 36.9)	27.6 (24.1, 31.6)

***Note***: Continuous variables are shown as median (25th, 75th percentile); categorical variables are shown are number (percentage).

*P<0.05 for facility versus home.

# P<0.05 for facility versus peritoneal dialysis versus home hemodialysis.

Patients may be classified in multiple modality categories due to multiple exposures over the duration of their follow-up.

**Figure 1:**
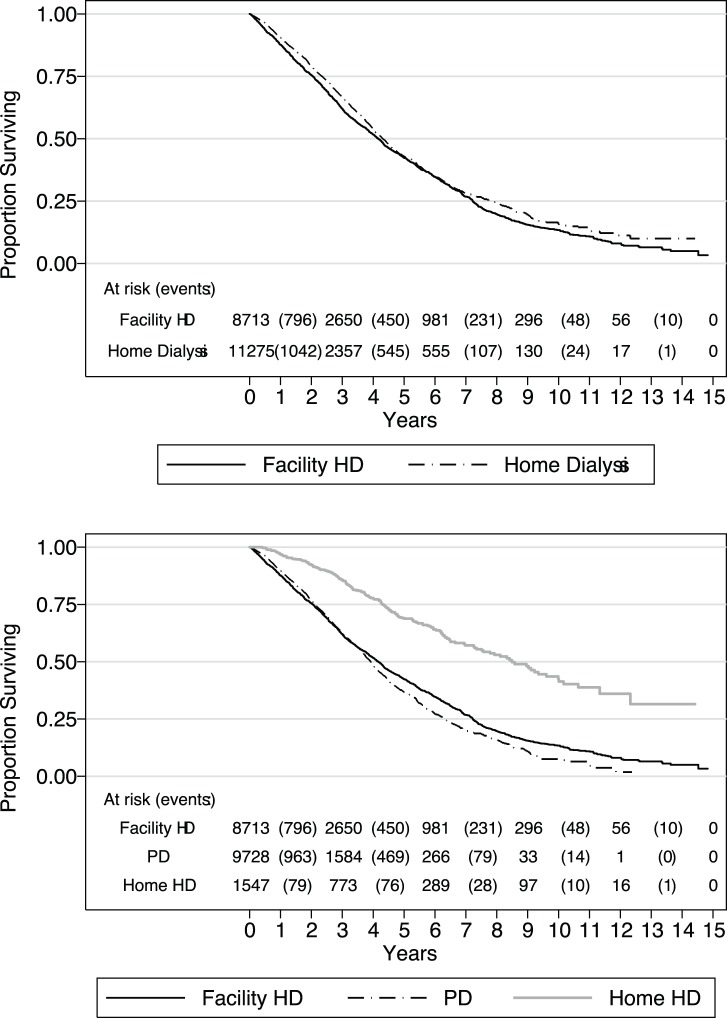
Kaplan Meier estimates of survival. Abbreviations: HD, haemodialysis; PD, peritoneal dialysis.

Kaplan Meier estimates of survival by modality are illustrated in Figure 1. The unadjusted median (interquartile range) survival for all patients in the study sample is 4.27 (4.12, 4.38) years. The corresponding values for those on facility HD and home dialysis are 4.16 (2.05, 7.23) and 4.30 (2.35, 7.79) years. The corresponding values for PD and home HD are 3.92 (2.11, 6.34) and 8.49 (4.30, −) years.

### Main Results

In the first set of analyses, we compared facility HD to home dialysis. In the second set of analyses, we compared facility HD with PD and home HD separately. All comparative results of mortality risk by modality are reported as hazard ratios relative to the reference category of facility HD.

**Figure 2:**
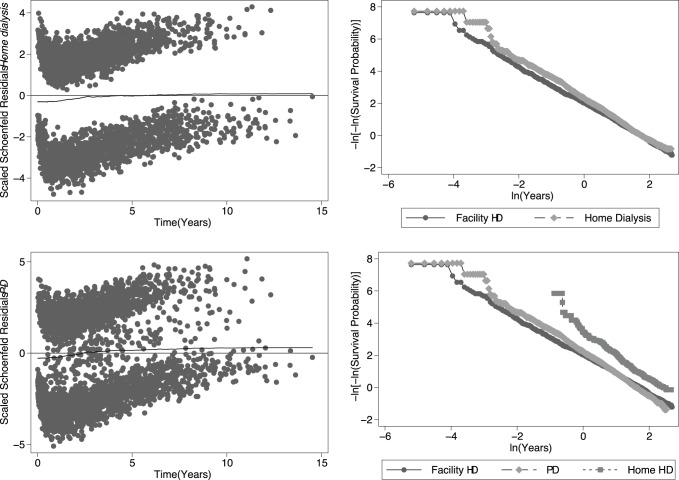
Time dependence of effects Illustrated using -ln [-ln(survival)] versus ln(analysis time) plots (left) and scaled Schoenfeld residuals plots (right). Plots for facility HD versus home dialysis are in the top panels, and for facility HD versus PD and home HD on the bottom.

Initial modelling demonstrated loss of proportional hazards for the variable home dialysis (P = 0.0021) and with the second set analyses confirming this for those on PD (P<0.00005) but not those treated with home HD (P = 0.36). Further modelling of the interaction between modality and time on dialysis show that both home dialysis and PD interacted with time at all levels up to and including 3 years (lower risk before, with higher risk of death after each time period compared to facility dialysis, see Table 3 and Figure 2). On this basis, we estimated main and interaction effects models for follow-up periods of <3 years (referred to hereafter as the “early” period) and >3 years (the “late” period).

**Table 3:** Time dependency of treatment effects, fully adjusted for the co-variates listed in Table 2.

Modality	Time Interaction Term	Time on Dialysis	Before	During	After
				Hazard ratio for mortality (95% CI)	
Home dialysis	Continuous/linear	1 year	0.68 (0.58–0.80)*		1.47 (1.23–1.47)**
	Continuous/linear	2 years	0.80 (0.70–0.87)*		1.33 (1.14–1.53)**
	Continuous/linear	3 years	0.81 (0.74–0.89)*		1.36 (1.17–1.57)**
	Category	0–1 years		1.0	
	Category	1–2 years		1.29 (1.00–1.61)#	
	Category	2–3 years		1.25 (0.98–1.57)	
	Category	≥3 years		1.57 (1.30–1.90)#	
Peritoneal dialysis	Continuous/linear	1 year	0.70 (0.60–0.82)*		1.64 (1.37–1.96)**
	Continuous/linear	2 years	0.83 (0.74–0.93)*		1.47 (1.27–1.71)**
	Continuous/linear	3 years	0.87 (0.79–0.96)*		1.54 (1.33–1.80)**
	Category	0–1 years		1.0	
	Category	1–2 years		1.35 (1.01–1.70)#	
	Category	2–3 years		1.32 (1.00–1.67)	
	Category	≥3 years		1.84 (1.51–2.24)#	

* P<0.05 versus facility HD.

** P<0.05 versus hazard ratio during earlier period.

# P<0.05 versus the baseline category of 0–1 years.

The interaction between modality and time is explored as a linear function of time on dialysis, and also a categorical one. Only results for home dialysis and PD are shown; home HD is not included since we did not identify any time dependency of effect. When modeling the treatment effect as a linear function of time, the point estimates in the later period indicate the additional change in effect during that period. For instance, the point estimates for home dialysis indicate a 19% reduction in mortality risk in the first 3 years, offset by an further 36% increase in relative risk in the period after three years for a total 10% increase in risk in the later period; explicitly, (1–1.36)*(1–0.81)+0.81 = 0.10. When modeling the treatment effect as a categorical function of time, the point estimates in the later period indicate the further change in effect during that period relative to the baseline category, in this case 0–1 years.

**Figure 3:**
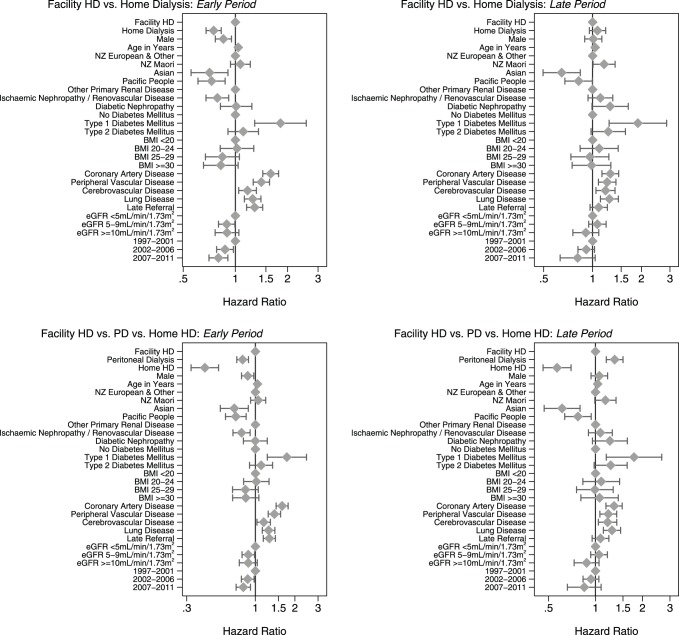
Hazard ratios for mortality from the main effects model comparing facility HD versus home dialysis (top panels) and facility HD versus PD and home HD (bottom panels), fully adjusted for the confounders listed in Table 2 (the markers represent point estimates, and the whiskers 95% confidence intervals). Estimates of effect for follow-up<3years are on the left (“Early period”); corresponding estimates for follow-up>3years are on the right (“Late period”). Abbreviations: BMI, body mass index; eGFR, estimated glomerular filtration rate; HD, haemodialysis; NZ, New Zealand; PD, peritoneal dialysis.

The results of the first set of analyses (comparing facility HD to home dialysis) are illustrated and tabulated in Figure 3 (top panels) and Table 4. Overall, there is a 13% lower mortality risk associated with home dialysis. However, the presence and extent of this effect varies over time: it is most marked in the early period and not apparent in the late period.

**Table 4:** Overall population conditional effects of modality on mortality, and interaction effects, fully adjusted for the co-variates in Table 2.

		Follow-up	Home Dialysis
			Hazard ratio for mortality (95% CI)
Total population		Overall	0.87 (0.81–0.94)*
		<3 years	0.75 (0.68–0.83)*
		>3 years	1.07 (0.96–1.21)
Comorbidity#	Absent	Overall	0.78 (0.67–0.90)*
		<3 years	0.64 (0.52–0.78)*
		>3 years	1.07 (0.85–1.35)
	Present	Overall	0.93 (0.85–1.01)
		<3 years	0.81 (0.72–0.91)*
		>3 years	1.08 (0.94–1.23)
Diabetes mellitus#	Absent	Overall	0.74 (0.66–0.83)*
		<3 years	0.65 (0.56–0.75)*
		>3 years	0.91 (0.77–1.09)
	Type 1	Overall	0.48 (0.29–0.78)*
		<3 years	0.38 (0.20–0.73)*
		>3 years	0.66 (0.32–1.33)
	Type 2	Overall	1.04 (0.94–1.16)
		<3 years	0.92 (0.80–1.05)
		>3 years	1.28 (1.10–1.50)*

*P values <0.05 relative to reference modality.

# P values <0.05 for interaction.

In the home dialysis, the primary exposure was defined as either PD or home HD. Facility HD is the reference modality.

The results of the second set of analyses (comparing facility HD with PD and home HD separately) are provided in Figure 3 (bottom panels) and Table 5. Overall, there is no difference in mortality risk between PD and facility HD, although there is a 20% lower mortality risk associated with PD in the early period that is offset by a 33% greater mortality risk in the late period. Overall, there is a 52% lower mortality risk associated with home HD, with no significant variation of the presence and extent of this effect over time.

**Table 5:** Overall population conditional effects of modality on mortality, and interaction effects, fully adjusted for the co-variates in Table 2.

			Peritoneal Dialysis	Home Hemodialysis
			Hazard ratio for mortality (95% CI)	
Total Population			0.98 (0.90–1.06)	0.48 (0.41–0.56)*
		follow-up<3 years	0.80 (0.72–0.88)*	0.41 (0.32–0.53)*
		follow-up>3 years	1.33 (1.17–1.50)*	0.57 (0.46–0.70)*
Ethnicity#	NZ European-&-Other	Overall	0.80 (0.71–0.90)*	0.34 (0.26–0.43)*
		follow-up<3 years	0.66 (0.57–0.76)*	0.28 (0.19–0.40)*
		follow-up>3 years	1.14 (0.93–1.38)	0.44 (0.31–0.61)*
	NZ Maori	Overall	1.15 (1.00–1.31)*	0.60 (0.48–0.76)*
		follow-up<3 years	1.04 (0.87–1.24)	0.61 (0.43–0.88)*
		follow-up>3 years	1.31 (1.07–1.61)*	0.60 (0.45–0.81)*
	Asian	Overall	1.00 (0.69–1.45)	0.61 (0.18–2.00)
		follow-up<3 years	0.72 (0.44–1.17)	0.54 (0.07–4.16)
		follow-up>3 years	1.29 (0.75–2.20)	0.45 (0.11–1.90)
	Pacific People	Overall	1.31 (1.06–1.63)*	0.81 (0.50–1.38)
		follow-up<3 years	1.01 (0.75–1.36)	0.91 (0.43–1.92)
		follow-up>3 years	1.87 (1.36–2.58)*	0.74 (0.35–1.57)
Comorbidity#	Absent	Overall	0.87 (0.74–1.02)	0.48 (0.37–0.63)*
		follow-up<3 years	0.68 (0.55–0.83)*	0.45 (0.30–0.66)*
		follow-up>3 years	1.37 (1.07–1.75)*	0.59 (0.40–0.86)*
	Present	Overall	1.02 (0.93–1.12)	0.48 (0.40–0.58)*
		follow-up<3 years	0.86 (0.76–0.96)*	0.39 (0.29–0.54)*
		follow-up>3 years	1.34 (1.16–1.54)*	0.57 (0.44–0.73)*
Diabetes mellitus#	Absent	Overall	0.85 (0.76–0.96)*	0.37 (0.29–0.47)*
		follow-up<3 years	0.69 (0.59–0.81)*	0.34 (0.24–0.49)*
		follow-up>3 years	1.16 (0.96–1.40)	0.41 (0.30–0.56)*
	Type 1	Overall	0.48 (0.29–0.80)*	0.44 (0.19–1.03)
		follow-up<3 years	0.42 (0.22–0.82)*	0.20 (0.04–0.90)*
		follow-up>3 years	0.62 (0.29–1.33)	0.92 (0.35–2.46)
	Type 2	Overall	1.15 (1.03–1.29)*	0.64 (0.52–0.80)*
		follow-up<3 years	0.95 (0.83–1.09)	0.60 (0.41–0.81)*
		follow-up>3 years	1.54 (1.30–1.82)*	0.70 (0.53–0.94)*
Year of dialysis Inception#	1997–2001	Overall	1.00 (0.87–1.13)	0.49 (0.39–0.62)*
		follow-up<3 years	0.71 (0.59–0.86)*	0.49 (0.33–0.71)*
		follow-up>3 years	1.43 (1.18–1.72)*	0.50 (0.36–0.68)*
	2002–2006	Overall	0.98 (0.87–1.11)*	0.51 (0.40–0.65)*
		follow-up<3 years	0.81 (0.69–0.96)*	0.43 (0.29–0.63)*
		follow-up>3 years	1.25 (1.04–1.49)*	0.60 (0.44–0.81)*
	2007–2011	Overall	0.86 (0.72–1.03)*	0.39 (0.26–0.62)*
		follow-up<3 years	0.79 (0.65–0.96)*	0.28 (0.16–0.50)*
		follow-up>3 years	1.47 (0.86–2.51)	0.96 (0.44–2.08)

*P values <0.05 relative to reference modality.

# P values <0.05 for interaction.

Conventional facility HD is the reference modality.

### Two-way and Three-way Interactions

In the first set of analyses, the effect of modality on mortality risk is not modified within subcategories of patient age, ethnicity, BMI, and year of dialysis inception. There is, however, notable modification of effect among patients with medical comorbidity. As per Table 4, the mortality risk associated with home dialysis and facility HD is similar in the early period in these groups, with an actual reversal of effect in those with type 2 diabetes mellitus in the late period: in these patients, there is a 28% higher mortality risk associated with home dialysis compared to facility HD. In the first set of analyses, there are no three-way interactions of statistical significance.

In the second set of analyses, the effect of modality on mortality risk is not modified within subcategories of patient age and BMI. There is minor modification of effect by the presence of medical comorbidity and by year of dialysis inception, although the results among subcategories of comorbidity and year are not materially different from those in the overall population. There is, however, notable modification of effect by ethnicity, and the presence and type of diabetes mellitus.

For PD, NZ European-&-Other patients as well as those without type 2 diabetes mellitus have a lower associated mortality risk compared to facility HD in the early period, and no difference in the late period. In NZ Maori and Pacific People and those with type 2 diabetes mellitus, there is no difference in mortality risk between PD and facility HD in the early period, and a higher mortality risk associated with PD in the late period.

For home HD, there is only minor modification of effect by most of these factors, although this may be due to the low statistical power in this group. Notwithstanding, there may be important modification of effect by ethnicity: in Pacific People, there is no difference in mortality risk between home HD and facility HD.

In the second set of analyses, there is a single three-way interaction of borderline statistical significance, between subcategories of ethnicity and year of dialysis inception. Exploration of this interaction within these subcategories produced results that are not materially different from those in the overall population (data not shown).

## Discussion

Our study generally supports the clinical and organizational culture of promoting home dialysis in NZ. Our main observations are from models that are fully adjusted for patient risk factors such as patient age and co-morbidity: 1) compared to facility HD, home dialysis is independently associated with improved overall survival; (2) compared to facility HD and PD, home HD is associated with the best overall survival; and (3) compared to facility HD, PD is associated with equivalent overall survival.

Our results comparing home HD and facility HD are consistent with those in the literature, including those found in a previous study of an Australian and NZ cohort: home HD has equivalent or better overall survival compared to facility HD and PD [4], [16]–[28]. We did not identify any modification of effect by time on dialysis or patient characteristics, with reasonable allowance for the reduced statistical power within this small group. In contrast, only some of our results comparing PD and facility HD are consistent with those in the literature, with particular differences around statistical interactions in our models. Traditionally, the effect of PD on survival is modified by time on dialysis [29]–[33], era [34], diabetes mellitus [4], [29]–[31], [33], [35]–[40], age [4], [29]–[31], [33], [35], [36], [40], BMI [32], [39], [41]–[43] and co-morbidity [37], [38]. The points of difference in our study are 1) the longer duration (3 years) of early relative survival advantage with PD than is usually described; 2) stable rather than improving relative survival with PD over time; 3) the absence of any modification of effect by age or BMI; and 4) the modification of effect by ethnicity.

The duration of early survival advantage with PD in our study was found to be longer than the 1–2 years usually identified by other investigators [29]–[32], and this finding persists in a variety of sensitivity analyses as shown in Table 2. The decreasing comparative survival with PD over time is usually attributed to diminishing residual renal function, changing peritoneal function with generally decreasing ultrafiltration capacity, and the use of central venous catheters early in the course of those starting dialysis with HD. It is likely that these factors are also present in our study population. The longer duration of early survival advantage in our study probably arises in part from the separation of home HD from facility HD in our analysis. This is different from the customary approach of all but one of the analysis in the literature, which traditionally compares PD with all HD, considering facility and home HD together irrespective of setting. Our approach allows for the correct handling of facility HD, modelling clinical outcomes that are more realistic than they might otherwise appear when facility HD is combined with home HD, which is the customary practice in most previous research. Our findings raise the possibility that the extent and duration of survival benefit associated with early PD may in fact be underestimated in the literature.

In our study, we were not able to identify secular improvements in relative survival over time. Such improvements have been noted for of PD patients in particular, albeit mostly in the United States where there is a particularly low prevalence of PD [44], [45]. It is likely that this has arisen from improvements to care over time, and such improvements are usually considered to include the introduction of disconnect systems, a shift from CAPD to APD, and the penetration of icodextrin and biocompatible dialysis solutions. There have been similar changes in NZ: the percentages of those APD, icodextrin and biocompatible dialysis solutions in close to zero in 1996 and 44%, 46%, 3% respectively in 2011. However, as stated, these changes have not been associated with the expected improvement in patient survival. This raises the possibility that improved outcomes for PD patients reported in the literature are less the result of technical advances, and perhaps more the result of increased experience and improved clinical care in regions where exposure to PD is low. In NZ, there is already longstanding experience with PD among the NZ nephrology community, and is likely to translate into stable practices based on a large amount of cumulative clinical knowledge and heuristics.

We did not identify any differences in early survival benefit associated with PD across subpopulations of patient age. In other studies in the literature, age probably functions as a marker of frail phenotype with increased (unmeasured) medical co-morbidity. We speculate that ethnicity may be functioning similarly in our study. However, ethnicity may also be exerting an effect through other unmeasured factors, such as socioeconomic status (lower status which is known to influence the technical success of dialysis at home [46], [47]), and also perhaps rurality (greater rurality is known to increase uptake of PD in those with poor access to facility dialysis and reluctance to relocate, irrespective of whether or not they are marginal or even frankly inappropriate for PD [48], [49]). Notwithstanding, the relatively poorer survival of NZ Maori and Pacific People on PD warrants further study, and perhaps an augmented clinical approach if findings are confirmed.

There will always be controversy as to the underlying reason for the better survival observed on home HD. On the one hand, there are no high quality (clinical trial) data showing that dialyses in the home environment in itself influences mortality. Using this reasoning, the observation of better survival should be ascribed to differences in the treatment itself (home HD being prescribed and delivered in a way that better manages fluid overload and uremia), or in the patients who undergo the treatment (home dialysis patients having unmeasured associated factors that result in better survival). On the other hand, there are consistent observational data showing better survival on home dialysis, with studies demonstrating that this is not entirely explained by age, measured co-morbidity, or the dialysis prescription [4]. Our own opinion is that there is a causal relationship between the home setting and better survival, probably mediated by greater adherence to dialysis, medication, fluid restriction, and lifestyle measures [7], [50]–[54]. It is plausible that these factors are also enablers to higher quality dialysis therapy, explaining the lower reported rates of technical and infectious events that is observed with home versus facility HD in the NZ setting [4], [55]. We are unable to explore these factors currently due to lack of appropriate data in the ANZDATA Registry. However, we are planning further analyses using database linkage to routinely collected NZ government datasets, with a view to reducing unmeasured confounding in our analyses by including domiciliary/social circumstances, socioeconomic deprivation, primary and secondary healthcare utilization, and medication prescription as co-variates. Notwithstanding, it is our opinion that the observed benefit of home HD arises in part from an enhanced culture of self-care with identified benefits as described.

Our results support an early survival advantage for PD, and the paradigm of a “PD first” policy which is under consideration in some parts of NZ. Our results also suggest the pressing need for study to determine optimal timing for switching modalities from PD to HD, which is surprisingly poorly researched given the importance of the issue. Timely transition from PD to HD in the late phase is associated with better survival, as is the avoidance of transition to PD after an unplanned start on HD in the early phase [56]–[58]. Our research suggests an appropriately powered randomized controlled trial, allocating patients to a switch from PD to HD at different pre-determined intervals, with appropriate collection of clinical and biochemical information from which to develop criteria and decision support systems.

As is the case for all observational studies, associations do not prove causality. Our study is likely to be limited by ascertainment error in the recording of co-morbidity, residual confounding from the limited collection of co-morbidity, and lack of socioeconomic, medication, and biochemical data in the analyses. Notwithstanding, our study supports the widespread adoption of home dialysis in NZ, and a drive to further increase home dialysis in that country. The immediate next steps for our research team include the following initiatives. We are planning further analyses in the NZ dialysis population that (i) incorporate additional measures of patients’ socioeconomic and functional status from data lonkage with government datasets, and (ii) address the variable and interchangeable exposure of patients in this country to HDF and frequent/extended HD during the course of their modality experience.

## References

[1] Lynn KL, Buttimore AL (2005) Future of home haemodialysis in Australia and New Zealand. Nephrology (Carlton) 10: 231–233. 15958034 10.1111/j.1440-1797.2005.00399.x

[2] McGregor D, Buttimore A, Robson R, Little P, Morton J, et al (2000) Thirty years of universal home dialysis in Christchurch. N Z Med J 113: 27–29. 11482325

[3] Blagg CR (2005) Home haemodialysis: ‘home, home, sweet, sweet home!’. Nephrology (Carlton) 10: 206–214. 15958031 10.1111/j.1440-1797.2005.00383.x

[4] Marshall MR, Hawley CM, Kerr PG, Polkinghorne KR, Marshall RJ, et al (2011) Home hemodialysis and mortality risk in Australian and New Zealand populations. Am J Kidney Dis 58: 782–793. 21816526 10.1053/j.ajkd.2011.04.027

[5] Wyld M, Morton RL, Hayen A, Howard K, Webster AC (2012) A systematic review and meta-analysis of utility-based quality of life in chronic kidney disease treatments. PLoS Med 9: e1001307. 22984353 10.1371/journal.pmed.1001307 PMC3439392

[6] Dale PL, Hutton J, Elgazzar H (2008) Utility of health states in chronic kidney disease: a structured review of the literature. Curr Med Res Opin 24: 193–206. 18039434 10.1185/030079908x253410

[7] Mowatt G, Vale L, MacLeod A (2004) Systematic review of the effectiveness of home versus hospital or satellite unit hemodialysis for people with end-stage renal failure. Int J Technol Assess Health Care 20: 258–268. 15446754 10.1017/s0266462304001060

[8] Mowatt G, Vale L, Perez J, Wyness L, Fraser C, et al (2003) Systematic review of the effectiveness and cost-effectiveness, and economic evaluation, of home versus hospital or satellite unit haemodialysis for people with end-stage renal failure. Health Technol Assess 7: 1–174. 10.3310/hta7020 12773260

[9] National Institutes of Health, National Institute of Diabetes and Digestive and Kidney Diseases (2012) U.S. Renal Data System, USRDS 2012 Annual Data Report: Atlas of Chronic Kidney Disease and End-Stage Renal Disease in the United States.

[10] Chan CT, Lok CE (2009) Why not home dialysis? Adv Chronic Kidney Dis 16: 158–159. 19393964 10.1053/j.ackd.2009.02.010

[11] Golper TA, Guest S, Glickman JD, Turk J, Pulliam JP (2011) Home Dialysis in the new USA Bundled Payment Plan: Implications and Impact. Peritoneal Dialysis International 31: 12–16. 21282384 10.3747/pdi.2010.00143

[12] Thodis ED, Oreopoulos DG (2011) Home dialysis first: a new paradigm for new ESRD patients. J Nephrol 24: 398–404. 21623574 10.5301/JN.2011.8374

[13] Vonesh E, Schaubel D, Hao W, Collins A (2000) Statistical methods for comparing mortality among ESRD pateints: Examples of regional/international variations. Kidney Int 57: S19–S27.

[14] The Australia and New Zealand Dialysis and Transplant Registry. Adelaide, Australia: ANZDATA Registry.

[15] Levey AS, Bosch JP, Lewis JB, Greene T, Rogers N, et al (1999) A more accurate method to estimate glomerular filtration rate from serum creatinine: a new prediction equation. Modification of Diet in Renal Disease Study Group. Ann Intern Med 130: 461–470. 10075613 10.7326/0003-4819-130-6-199903160-00002

[16] Mailloux LU, Bellucci AG, Mossey RT, Napolitano B, Moore T, et al (1988) Predictors of survival in patients undergoing dialysis. American Journal of Medicine 84: 855–862. 3364444 10.1016/0002-9343(88)90063-0

[17] Mailloux LU, Bellucci AG, Napolitano B, Mossey T, Wilkes BM, et al (1994) Survival estimates for 683 patients starting dialysis from 1970 through 1989: Identification of risk factors for survival. Clinical Nephrology 42: 127–135. 7955575

[18] Mailloux LU, Kapikian N, Napolitano B, Mossey RT, Bellucci AG, et al (1996) Home hemodialysis: patient outcomes during a 24-year period of time from 1970 through 1993. Adv Ren Replace Ther 3: 112–119. 8814916 10.1016/s1073-4449(96)80050-1

[19] Prowant B, Nolph KD, Dutton S, et al (1983) Actuarial analysis of patient survival and dropout with various end-stage renal disease therapies. American Journal of Kidney Diseases 3: 27–31. 6346864 10.1016/s0272-6386(83)80006-7

[20] Saner E, Nitsch D, Descoeudres C, Frey FJ, Uehlinger DE (2005) Outcome of home haemodialysis patients: a case-cohort study. Nephrol Dial Transplant 20: 604–610. 15665030 10.1093/ndt/gfh674

[21] Weller JM, Port FK, Swartz RD, Ferguson CW, Williams GW, et al (1982) Analysis of survival of end-stage renal disease patients. Kidney Int 21: 78–83. 7043052 10.1038/ki.1982.11

[22] Williams GW, Weller JM, Ferguson CW, Forsythe SB, Wu SC (1983) Survival of endstage renal disease patients: age-adjusted differences in treatment outcomes. Kidney Int 24: 691–693. 6363799 10.1038/ki.1983.212

[23] Woods JD, Port FK, Stannard D, Blagg CR, Held PJ (1996) Comparison of mortality with home hemodialysis and center hemodialysis: a national study. Kidney Int 49: 1464–1470. 8731115 10.1038/ki.1996.206

[24] Rubin J, Barnes T, Bower J (1985) Morbidity and mortality in CAPD and home hemodialysis: One Center’s five-year experience. ASAIO Journal 8: 22–27.

[25] Rubin J, Barnes T, Burns P, et al (1983) Comparison of home hemodialysis to continuous ambulatory peritoneal dialysis. Kidney International 23: 51–56. 6834694 10.1038/ki.1983.10

[26] Rubin J, Hsu H, Bower J (1989) Survival on dialysis therapy: One center’s experience. American Journal of the Medical Sciences 297: 80–90. 2919635 10.1097/00000441-198902000-00004

[27] Grant AC, Rodger RSC, Howie CA, Junor BJR, Briggs JD, et al (1992) Dialysis at home in the west of Scotland: A comparison of hemodialysis and continuous ambulatory peritoneal dialysis in age- and sex-matched controls. Peritoneal Dialysis International 12: 365–368. 1420494

[28] Nitsch D, Steenkamp R, Tomson CR, Roderick P, Ansell D, et al (2011) Outcomes in patients on home haemodialysis in England and Wales, 1997–2005: a comparative cohort analysis. Nephrol Dial Transplant 26: 1670–1677. 20841489 10.1093/ndt/gfq561

[29] Heaf JG, Lokkegaard H, Madsen M (2002) Initial survival advantage of peritoneal dialysis relative to haemodialysis. Nephrol Dial Transplant 17: 112–117. 11773473 10.1093/ndt/17.1.112

[30] Termorshuizen F, Korevaar JC, Dekker FW, Van Manen JG, Boeschoten EW, et al (2003) Hemodialysis and peritoneal dialysis: comparison of adjusted mortality rates according to the duration of dialysis: analysis of The Netherlands Cooperative Study on the Adequacy of Dialysis 2. J Am Soc Nephrol 14: 2851–2860. 14569095 10.1097/01.asn.0000091585.45723.9e

[31] Jaar BG, Coresh J, Plantinga LC, Fink NE, Klag MJ, et al (2005) Comparing the risk for death with peritoneal dialysis and hemodialysis in a national cohort of patients with chronic kidney disease. Ann Intern Med 143: 174–183. 16061915 10.7326/0003-4819-143-3-200508020-00003

[32] McDonald SP, Marshall MR, Johnson DW, Polkinghorne KR (2009) Relationship between dialysis modality and mortality. J Am Soc Nephrol 20: 155–163. 19092128 10.1681/ASN.2007111188 PMC2615722

[33] Schaubel DE, Morrison HI, Fenton SS (1998) Comparing mortality rates on CAPD/CCPD and hemodialysis. The Canadian experience: fact or fiction? Perit Dial Int 18: 478–484. 9848625

[34] Vonesh EF, Snyder JJ, Foley RN, Collins AJ (2004) The differential impact of risk factors on mortality in hemodialysis and peritoneal dialysis. Kidney Int 66: 2389–2401. 15569331 10.1111/j.1523-1755.2004.66028.x

[35] Vonesh EF, Snyder JJ, Foley RN, Collins AJ (2006) Mortality studies comparing peritoneal dialysis and hemodialysis: what do they tell us? Kidney Int Suppl: S3–11. 10.1038/sj.ki.5001910 17080109

[36] Collins AJ, Hao W, Xia H, Ebben JP, Everson SE, et al (1999) Mortality risks of peritoneal dialysis and hemodialysis. Am J Kidney Dis 34: 1065–1074. 10585316 10.1016/S0272-6386(99)70012-0

[37] Ganesh SK, Hulbert-Shearon T, Port FK, Eagle K, Stack AG (2003) Mortality differences by dialysis modality among incident ESRD patients with and without coronary artery disease. J Am Soc Nephrol 14: 415–424. 12538742 10.1097/01.asn.0000043140.23422.4f

[38] Stack AG, Molony DA, Rahman NS, Dosekun A, Murthy B (2003) Impact of dialysis modality on survival of new ESRD patients with congestive heart failure in the United States. Kidney Int 64: 1071–1079. 12911559 10.1046/j.1523-1755.2003.00165.x

[39] Stack AG, Murthy BV, Molony DA (2004) Survival differences between peritoneal dialysis and hemodialysis among “large” ESRD patients in the United States. Kidney Int 65: 2398–2408. 15149353 10.1111/j.1523-1755.2004.00654.x

[40] Winkelmayer WC, Glynn RJ, Mittleman MA, Levin R, Pliskin JS, et al (2002) Comparing mortality of elderly patients on hemodialysis versus peritoneal dialysis: a propensity score approach. J Am Soc Nephrol 13: 2353–2362. 12191980 10.1097/01.asn.0000025785.41314.76

[41] Abbott KC, Glanton CW, Trespalacios FC, Oliver DK, Ortiz MI, et al (2004) Body mass index, dialysis modality, and survival: analysis of the United States Renal Data System Dialysis Morbidity and Mortality Wave II Study. Kidney Int 65: 597–605. 14717930 10.1111/j.1523-1755.2004.00385.x

[42] Snyder JJ, Foley RN, Gilbertson DT, Vonesh EF, Collins AJ (2003) Body size and outcomes on peritoneal dialysis in the United States. Kidney Int 64: 1838–1844. 14531819 10.1046/j.1523-1755.2003.00287.x

[43] Inrig JK, Sun JL, Yang Q, Briley LP, Szczech LA (2006) Mortality by dialysis modality among patients who have end-stage renal disease and are awaiting renal transplantation. Clin J Am Soc Nephrol 1: 774–779. 17699286 10.2215/CJN.00580705

[44] Churchill DN, Thorpe KE, Vonesh EF, Keshaviah PR (1997) Lower probability of patient survival with continuous peritoneal dialysis in the United States compared with Canada. Canada-USA (CANUSA) Peritoneal Dialysis Study Group. J Am Soc Nephrol 8: 965–971. 9189865 10.1681/ASN.V86965

[45] Vonesh EF, Moran J (1999) Mortality in end-stage renal disease: a reassessment of differences between patients treated with hemodialysis and peritoneal dialysis. J Am Soc Nephrol 10: 354–365. 10215336 10.1681/ASN.V102354

[46] Sanabria M, Munoz J, Trillos C, Hernandez G, Latorre C, et al.. (2008) Dialysis outcomes in Colombia (DOC) study: a comparison of patient survival on peritoneal dialysis vs hemodialysis in Colombia. Kidney Int Suppl: S165–172. 10.1038/sj.ki.5002619 18379541

[47] Xu R, Han Q-F, Zhu T-Y, Ren Y-P, Chen J-H, et al (2012) Impact of Individual and Environmental Socioeconomic Status on Peritoneal Dialysis Outcomes: A Retrospective Multicenter Cohort Study. PLoS ONE 7: e50766. 23226378 10.1371/journal.pone.0050766 PMC3511320

[48] Rodriguez RA (2012) Dialysis and mortality: does it matter where you live? Clin J Am Soc Nephrol 7: 1055–1057. 22723448 10.2215/CJN.05410512

[49] Tonelli M, Hemmelgarn B, Culleton B, Klarenbach S, Gill JS, et al (2007) Mortality of Canadians treated by peritoneal dialysis in remote locations. Kidney Int 72: 1023–1028. 17637709 10.1038/sj.ki.5002443

[50] Christensen AJ, Smith TW, Turner CW, Holman JM Jr, Gregory MC (1990) Type of hemodialysis and preference for behavioral involvement: interactive effects on adherence in end-stage renal disease. Health Psychol 9: 225–236. 2331980 10.1037//0278-6133.9.2.225

[51] Kraus M, Burkart J, Hegeman R, Solomon R, Coplon N, et al (2007) A comparison of center-based vs. home-based daily hemodialysis for patients with end-stage renal disease. Hemodial Int 11: 468–477. 17922746 10.1111/j.1542-4758.2007.00229.x

[52] Polaschek N (2007) Client attitudes towards home dialysis therapy. Edtna-Erca Journal 33: 20–24. 10.1111/j.1755-6686.2007.tb00032.x 17695557

[53] Christensen AJ, Smith TW, Turner CW, Holman JM Jr, Gregory MC, et al (1992) Family support, physical impairment, and adherence in hemodialysis: an investigation of main and buffering effects. J Behav Med 15: 313–325. 1404348 10.1007/BF00844725

[54] Snyder WL (1977) Factors affecting adherence to diet and medication orders by hemodialysis patients. Abstracts of Hospital Management Studies 14: 18102 PA: 18167p.

[55] McGregor DO, Buttimore AL, Lynn KL, Nicholls MG, Jardine DL (2001) A comparative study of blood pressure control with short in-center versus long home hemodialysis. Blood Purification 19: 293–300. 11244189 10.1159/000046957

[56] Lameire N, Van Biesen W, Vanholder R (2000) The role of peritoneal dialysis as first modality in an integrative approach to patients with end-stage renal disease. Perit Dial Int 20 Suppl 2S134–141. 10911659

[57] Van Biesen W, Vanholder RC, Veys N, Dhondt A, Lameire NH (2000) An evaluation of an integrative care approach for end-stage renal disease patients. J Am Soc Nephrol 11: 116–125. 10616847 10.1681/ASN.V111116

[58] Boissinot L, Landru I, Cardineau E, Zagdoun E, Ryckelycnk JP, et al (2013) Is transition between peritoneal dialysis and hemodialysis really a gradual process? Perit Dial Int 33: 391–397. 23284075 10.3747/pdi.2011.00134 PMC3707717

